# Factors associated with the achievement of cervical smears by general practitioners

**DOI:** 10.1186/s13104-017-2999-5

**Published:** 2017-12-08

**Authors:** Michaël Rochoy, Thibaut Raginel, Jonathan Favre, Estelle Soueres, Nassir Messaadi, Valérie Deken, Alain Duhamel, Christophe Berkhout

**Affiliations:** 1 Department of General Practice/Family Medicine, School of Medicine, Lille University UDSL, 59000 Lille, France; 2 UCBN, INSERM U1086, Cancers & Preventions, CHU de Caen, Normandie University, 14000 Caen, France; 3 UCBN, Department of General Medicine, Medical School, Normandie University, 14000 Caen, France; 4 Department of Public Health, University Hospital Lille, Lille University EA 2694, 59000 Lille, France; 5 Department of General Practice/Family Medicine, School of Medicine, Lille University-UDSL, 59045 Lille Cedex, France

**Keywords:** Vaginal smears, Uterine cervical neoplasms, Early detection of cancer, Mass screening, Physicians, Primary care

## Abstract

**Objective:**

Reliable data about general practitioners performing pap-tests are insufficient. A claim code for the achievement of pap-smears exists in France, but its use by general practitioners is not known. The main purpose of this study was to highlight independent factors associated with the achievement of pap-smears by the general practitioner (GP). We carried out a descriptive and analytic epidemiologic study in 347 GPs and their 244,889 patients, registered at the Health Care Insurance Fund of Flanders. The European Deprivation Index (EDI) in the area of GP’s surgeries was specified. All GPs were questioned by telephone about their performance of pap-tests. The claim database of the insurance fund was analyzed to describe characteristics of GPs.

**Results:**

The answer rate among questioned GPs was 98.8%. Pap-smears were performed in their surgeries by 182 GPs (53.1%). Among males, 45.7% performed pap-smears versus 78.4% of the female (adjusted odds-ratio = 4.5, p < 0.001). The mean rate of screened women in the target population was 44% when GPs were performing smears versus 42% when they were not (adjusted odds-ratio = 1.04, p = 0.03). Only 19.5% of GPs used the claim code. The number of patients, and the EDI were not associated with pap-smears.

*Trial registration* ClinicalTrials.gov NCT02749110 (April 22, 2016)

## Introduction

In France, cervical cancer was the eleventh most frequent cancer in women in 2012 (3028 cases and 1102 allocated deaths) [1]. According to the French National Authority for Health (*Haute autorité de santé*, HAS), guidance recommends screening for cervical cancer basing on a 3-year interval cervical smear, from 25 to 65 years of age, after 2 normal initial yearly smears [2]. In France, in 2010, the cervical cancer screening coverage was suboptimal: more than half of French women were under- or unscreened [3]. In 2016, there was still no national organized (systematic) cervical cancer screening program: screening remains opportunistic [4, 5]. However, 13 counties (*départements*) out of 100 implement organized screening [6].

The pap-test (or Papanicolaou-test or smear-test) consists in collecting shedding cells at the outer orifice of the cervix for a cytological examination to detect neoplasms caused by a persisting human papillomavirus infection. Sampling can be carried out in France by medical doctors [as general practitioners (GPs), gynecologists, or medical biologists] and midwives. Though about half of GPs achieve pap-smears in their surgeries, the percentage of smears that are performed by GPs range from 5 to 10% [7, 8].

Data concerning GPs’ uses regarding cervical smear performance in countries with opportunistic screening are scarce in literature. As far as we know, no study evaluating pap-smear coding by GPs was performed in France: the use of this code (JKHD001) is to claim an extra fee (€.6.23) for the performance of the pap-smear in the French fee for service payment system.

The aim of this study was to seek for independent factors associated with the performance of pap-smears by the GP and the reliability of the data provided by the SIAM-ERASME registry of the Health Care Insurance Fund (*Caisse primaire d’assurance maladie,* CPAM) of (French) Flanders. This information, not provided by the literature, was a necessary preamble to design properly the stratified cluster randomization of the PaCUDAHL-Gé cervical cancer screening trial to insure its external validity.

## Main text

### Methods

This descriptive and analytic epidemiologic study was carried out between March 1 and June 22, 2015, basing on the SIAM-ERASME claim database of the CPAM of Flanders (France). We enrolled all practicing GPs registered at the CPAM of Flanders on March 1, 2015. We excluded retired physicians and GPs having less than 100 females on their patients’ lists, in order to eliminate recently settled physicians or GPs with a peculiar practice (i.e. homeopathy, acupuncture, angiology, etc.).

The main outcome was the performance of cervical smears. In order to maximize the response rate, 20 GP registrars (residents) interviewed the enrolled GPs by telephone asking a single binary question: “*Do you perform any cervical smear in your surgery?*”

The explicative variables were physicians’ features: gender, number of listed patients (i.e. patients who declared at the CPAM they were managed by this GP), number of listed female patients, number of listed female patients between 25 and 65 years of age, number of female patients from 25 to 65 for whom a pap-test cytology had been refunded by the CPAM during the past 2 years (basing on cytology claim codes), and at least one declared pap-test claim code (JKHD001). One additional variable associated to the deprivation index of the geographic area of GPs’ surgeries was recorded: the European Deprivation Index (EDI), computed from the last population census (2007) [9].

In descriptive analysis, the quantitative variables are expressed as means, medians, standard deviations, first and third quartile and range. The qualitative variables are expressed as the frequencies and percentages. In this study, there were two levels for the data: the individual level (GP’s characteristics and the main outcome “achievement of pap-tests”) who were nested in the geographical level (variable EDI). All analyses were performed by using a hierarchical generalized mixed model. This statistical model allows performing a logistic regression taking into account the hierarchical structure of data. The association between each individual characteristic of GPs was first analyzed separately with an adjustment on EDI. The GPs’ level variables having a p value less than 0.2 were introduced in a multivariate analysis (adjusted for EDI) in order to identify the subset of variables linked to the main outcome (achievement of pap-tests). The odds ratios (OR) with 95% confidence intervals (95% CI) were computed instead of prevalence rates that are not applicable to the hierarchical structure of the individual level of variables nested in the geographical level. The association between the EDI and the main outcome was also assessed using the generalized linear model. The significant level for the tests was 0.05. Statistical testing was done at the two-tailed α level of 0.05. All statistical analyses were performed by the statistics team of the CHRU of Lille, using the SAS software version 9.3.

This study is a preliminary study to the PaCUDAHL-Gé trial. PACUDAHL-Gé is promoted by the University Hospital of Lille and funded by the French Ministry of Health (PREPS: LIC-14-14-0615). Agreement was gathered from the French Agency for the Safety of Health Products (ANSM: 2015-A01331-48) and the Ethics Committee (CPP Nord-Ouest III: 2015-23). The protocol is searchable on ClinicalTrials.gov (NCT02749110). None of the authors have any competing interest.

### Results

On March 1st, 2015, 402 GPs were registered at the CPAM of Flanders; 50 were excluded because they had less than 100 listed female patients and five for retirement. The total number of listed patients of the remaining 347 GPs was 244,889, corresponding to 131,053 female patients and 90,094 females between 25 and 65 years of age (cervical cancer screening target). Among these, 38,070 had a pap-test cytology refunded by the CPAM during the past 2 years.

Of the 347 eligible GPs, 343 answered the investigators’ question (answer rate: 98.8%) (Table 1).

**Table 1:** Study GPs’ characteristics

Characteristics (N = 343)	
Gender	
Female	74 (21.6%)
Number of listed patients	
Mean (SD)	698 (290)
Median (Q1–Q3)	668 (491 to 852)
Range	139 to 1845
Number of female listed patients	
Mean (SD)	374 (157)
Median (Q1–Q3)	352 (264 to 450)
Range	100 to 1104
Number of female listed patients, aged 25–65 and screened (whoever operates the pap-smear)	
Mean (SD)	43 (7)
Median (Q1–Q3)	44 (39; − 48)
Range	20 to 62
European Deprivation Index (EDI) (N = 341)	
Mean (SD)	2.2 (3.7)
Median (Q1–Q3)	1.9 (− 0.6 to 3.7)
Range	− 4.3 to 15.6
Rating of pap-smear	
Number of GPs (%)	67 (19.5%)

*SD* standard deviation, *Q1–Q3* interquartile interval, *%* percent, *EDI* European Deprivation Index

Of these 343 GPs, 182 (53.1%) declared performing cervical smears, 45.7% in male GPs and 79.7% in female, and 60 (33%) used the claim code JKHD001 (19.8% of all GPs). EDI was not found for 2 GPs due to an administrative area redrawing since 2007.

The EDI index was not significantly associated to the realization of pap-smears by GPs (OR: 0.95; 95% CI 0.89–1.02; 2.0 ± 3.6 versus 2.6 ± 3.8 respectively, *p* = 0.16). Using bivariate analysis adjusted for EDI, the total number of listed patients for each GP and the total number of listed female patients does not appear to be associated to the performance of pap-smears by the GP. Multivariate analysis showed a significant association between the performance of pap-tests by the GPs and GPs’ gender (*p* < 0.001) and the rate of female patients screened in the target population (whoever performed the pap-smear: GP, gynecologist, biologist or midwife) (*p* = 0.03) (Table 2).

**Table 2:** Characteristics of the 343 GPs and their patients basing on their achievement of pap-smear; variables associated with the performance of pap-smears by the GPs

Characteristics	Pap-smear: no (n = 161)	Pap-smear: yes (n = 182)	Bivariate analysis		Multivariate analysis	
			OR (95% CI)	p value (adjusted on EDI)	OR (95% CI)	p value (adjusted on EDI)
Gender: female, N (%)	15 (9.3%)	59 (32.4%)	4.8920 (2.5491–9.3883)	< 0.001	4.5 (2.3–8.7)	< 0.001
Number of listed patients, mean (SD)	728 (297)	671 (281)	0.9994 (0.9982–1.0005)	0.27		
Number listed female patients, mean (SD)	381 (156)	367 (157)	0.9997 (0.9975–1.0018)	0.75		
Participation rate of screened female patients aged 25–65, mean (SD)	42.1 (7.0)	44.4 (6.7)	1.0523 (1.0142–1.0919)	*0.007*	1.04 (1.0–1.08)	*0.03*

*SD* standard deviation, *NA* not applicable, *OR* odds ratio, *95% CI* 95% confidence interval, *EDI* European Deprivation Index

### Discussion

Basing on 347 practicing GPs registered at the CPAM of Flanders and their 90,094 female patients eligible for cervical cancer screening, factors associated with the performance of pap-smears by the GP were the female gender of the GP and the rate of screened women in the target population (whoever operated the pap-test). The total number of listed patients, the number of listed female patients and the deprivation index of the surgery’s area were not associated with the achievement of pap-tests by the GP. Among GPs, 53% were carrying out pap-tests, but only 20% of all GPs were using the claim code. Coding pap-smears is rather recent for French GPs, and it is of interest to follow-up this variable to find out if any improvement of this proportion is noted.

In a study conducted among GPs in Brittany in 2005 with a similar sex-ratio, 37% of them declared performing more than 12 gynecologic encounters per week [10]. A survey conducted in Burgundy in 2004 gathering 192 GPs found that 73% of them declared having been performing a pap-smear to their last patient aged between 25 and 65 years [11]. Amidst 852 GPs from a survey conducted in Essonne (southern suburb of Paris), 46% of respondents reported performing smears, which is a similar finding as ours [3].

In our study, 43% of female listed patients in the target age interval were screened by pap-smear over 2 years, regardless of the operator. Between 2006 and 2008, cervical smear coverage over 3 years was 56.6% [3]. Considering one missing year, this major difference is a characteristic of the French Flanders area, chosen because being one where the screening participation rate is the lowest in France and where the incidence of cervical cancer is one of the highest. The PACUDAHL-Gé population is socio-economically more deprived than the French mean population—socio-economic deprivation being clearly identified as one of the main factors of low screening participation rates [12, 13]. This weak participation rate increases the chance to highlight a statistical significance in the PaCUDAHL-Gé trial [14].

If attendance to cervical cancer screening is lower in deprived areas, it is not due to health service development issues. Indeed, there was no association found between the setting of GP surgeries in deprived areas measured by EDIs and performance of cervical smears by GPs or density of gynecologists in the area.

We could not find in the literature any data regarding cervical smear coding by GPs, in France or in other countries. We report here a significant discrepancy between the rating and the performance of pap-smears, which means that assessments of pap-smears performance by GPs basing on pap-smears’ rating is not reliable in the SIAM claim database and is under-estimated by about 60%.

We highlighted an association between female GPs and the performance of cervical smears. This finding is not new, and this association is widely reported in literature, with a higher coverage of all primary health care procedures directed towards female patients, in particular pap-smears [15–19]. In a survey study carried out in Burgundy in 2004, 87.5% of female GPs declared they had performed a pap-smear to their last female patient aged from 25 to 65 years, versus 68.8% of the males (*p* < 0.05) [11]. These results are higher than ours (respectively 79.7 and 45.7%) but are prone to memory and selection bias.

We haven’t found any association between the number of listed patients and the performance of pap-smears. This matches with Webb et al. who demonstrated that an increasing number of listed patients didn’t minimize the performance of pap-smears by GPs [20]. In the same way, Bang et al. found that the number of female patients listed by GP was not linked to cervical cancer screening participation [12].

## Limitations

One of the main strengths of our study is the size of the sample, which almost included the entire GP-population of a district, and the extremely high participation rate. These outcomes resulting from GPs’ interviews reflect accurately GPs’ practices, without selection bias. Another strength is the use of the comprehensive SIAM database of the CPAM of Flanders: it allowed an access to validated and structured information [21]. We used the EDI as adjustment variable: this validated environmental factor reflects confusion factors related to socioeconomic resources in diverse GP surgeries’ settings.

This study also has its limits. It was carried out among the GP population registered at the CPAM of Flanders, and its outcomes do not necessarily reflect the situation of the rest of France. When GPs were contacted, they were only asked for a binary answer about their cervical smear performance in way to maximize the response rate. Those carrying out very few smears (in particular, those performing smears only in patients insistently asking for) answered accordingly to their attitudes regarding their way to practice: sometimes yes, sometimes no. But most physicians who regularly carried out smears and those who never did gave clear answers. Ambiguous answers, if they might have masked any associations, never challenged the highlighted associations.

The data from the CPAM of Flanders we used covered 2 years and not 3. Yet, screening by pap-tests is recommended every 3 years in France. One year is missing to compute screening participation rates according to the French guidelines. Nevertheless, this fact does not affect the validity of the demonstrated associations or the inexistent links. Furthermore, the purpose of this study was not to compute the screening rate, but to screen variables allowing the stratification of the cluster randomization of GP investigators for the PaCUDAHL-Gé trial.

## Abbreviations

95% CI: 95% confidence interval
CPAM: Health Care Insurance Fund (*Caisse primaire d’assurance maladie*)
EDI: European Deprivation Index
GP: general practitioner
HAS: French National Authority for Health (*Haute autorité de santé*)
OR: odds ratio
